# Validation and Clinical Application of a Developed Pedal Coordination Assessment Device for Older Drivers

**DOI:** 10.3390/healthcare13050537

**Published:** 2025-03-01

**Authors:** Tsutomu Sasaki, Kyohei Yamada, Yoshio Tsuchiya, Moeka Takimoto

**Affiliations:** 1 Department of Rehabilitation, School of Health Sciences, Hokkaido Chitose College of Rehabilitation, Chitose 066-0055, Japan; k-yamada@chitose-reha.ac.jp; 2 Faculty of Engineering and Design, Kagawa University, Takamatsu 761-0396, Japan; tsuchiya.yoshio@kagawa-u.ac.jp; 3 National Institute of Technology, Tomakomai College, Tomakomai 059-1275, Japan; tm20114@stu.tomakomai-ct.ac.jp

**Keywords:** older drivers, driving ability, pedal coordination, physical function, cognitive function, on-road test, driving simulator

## Abstract

**Background**: Automobiles are vital for older adults to maintain health and independence, yet aging-related declines in physical and cognitive functions may impair driving abilities. Compensatory driving behaviors can help maintain driving safety. **Objectives**: This study aims to (1) validate a newly developed pedal coordination assessment device reflecting aging effects and (2) examine its potential application by analyzing the relationship between pedal coordination ability, physical and cognitive functions, and driving abilities. **Methods**: A total of 48 older adults (mean age 73.5 ± 4.9 years) and 56 younger adults (mean age 21.1 ± 0.7 years) participated. Older adults underwent on-road evaluations, driving simulator (DS) tests, and assessments of grip strength, Short Physical Performance Battery (SPPB), Mini-Mental State Examination (MMSE), and Trail Making Test. Pedal coordination ability was measured using the device, requiring participants to maintain pedal positions across target speeds. Correlations between pedal coordination ability and physical, cognitive, and driving-related indicators were analyzed. **Results**: Older adults required significantly more time than younger adults to maintain pedal positions. Within older adults, women showed poorer performance than men. Limited correlations were found between pedal coordination ability and physical or cognitive functions. Positive correlations were observed with DS accident frequency and sudden braking incidents. **Conclusions**: The device effectively reflected age-related declines in pedal coordination ability. Its primary application should focus on helping older adults recognize aging-related changes and promoting compensatory driving behaviors. Future studies should explore its use in group-based preventive programs.

## 1. Introduction

According to statistics from the World Health Organization [1], the number of fatalities caused by automobile accidents reaches 1.2 million annually, with approximately 30% of these fatalities involving individuals aged 65 and older. Traffic accidents are influenced by human factors [2], environmental factors [3], and vehicle-related factors [4]. Therefore, achieving the WHO’s goal of “Reducing traffic accident fatalities and injuries by 50% by 2030” [5] requires collaborative efforts from various professions and industries to mitigate traffic accidents. As healthcare professionals, we have a particular responsibility to contribute to the reduction of human factors. To reduce accidents involving older adults by addressing human factors, various interventions have been implemented worldwide, including physical training [6], cognitive training [7], visual/perceptual training [8], safe driving education [9], and combined training programs incorporating multiple approaches [10]. While individual training programs alone may have limited impact, combined training has been recommended as an effective approach for reducing traffic accidents [11].

For older adults, automobiles are an essential means of transportation to maintain health and independent living. Physical and cognitive decline with age may impair driving ability. However, diminished driving ability does not necessarily lead to unsafe driving. By adopting self-regulatory driving practices such as avoiding speeding, refraining from driving at night or in adverse weather conditions, and engaging in self-regulatory driving practices, older adults can maintain their ability to drive safely [12]. The finding that older adults have lower accident rates than younger drivers implicitly supports this hypothesis [13]. To promote self-regulatory driving, it is essential for older drivers to undergo professional evaluation and become aware of any decline in their driving abilities or the physical and cognitive functions necessary for driving [14,15,16]. Representative driving ability assessments include on-road driving evaluations, driving simulators (DS) [17], pedal response tests [18], hazard perception tests [19], the Useful Field of View (UFOV) test [20], and the Trail Making Test (TMT) [21]. Among these, on-road driving evaluations, DS, and pedal response tests are particularly effective, as they mimic actual driving operations and situations, allowing participants to easily reflect on their driving ability. However, the implementation of on-road driving evaluations, DS, and pedal response tests poses significant barriers for older adults. These assessments require specialized equipment or facilities and face-to-face individual evaluations and are often costly. To mitigate these challenges, we developed a pedal adjustment ability assessment device that is free and suitable for group-based evaluation. The intended application of this device is in group-participatory caregiving prevention programs that Japanese municipalities are mandated to implement. Because these programs are funded by local governments, participants incur no financial burden.

In several countries, initiatives have been implemented to support older adults through vehicle modifications and safe driving advice [22], as well as assistance in maintaining mobility through driving and alternative transportation options [23]. The former is a program based on individualized assessments, while the latter is a form of proactive education aimed at reducing decision conflict when ceasing driving and promoting health maintenance after driving cessation. One of the novel aspects of the preventive care program implemented by the authors is its group-based approach. This program aims to enhance older adults’ awareness of their declining driving ability and the physical and cognitive functions necessary for driving, thereby promoting self-regulatory driving.

The objectives of this study were twofold. The first was to validate the developed pedal coordination assessment device. Previous studies have shown that older adults exhibit a decline in pedal operation ability compared with younger adults [24,25]. If the developed device accurately reflects the effects of aging, the performance of older adults should be inferior to that of younger adults. The second objective is to explore the potential applications of the pedal coordination assessment device. To this end, we analyzed the relationships among pedal coordination ability, physical and cognitive functions, and driving ability in older adults. Depending on the identified associations, the application of this device in preventive care programs may vary.

## 2. Materials and Methods

### 2.1. Participants

Older adults were recruited verbally from participants in a preventive care program in which the authors were involved. The study details were explained to those who expressed interest in both written and verbal formats, and informed consent was obtained. Participants were included if they were 60 years of age or older and possessed a valid driver’s license. Individuals with a Mini-Mental State Examination (MMSE) [26] score of 23 or lower, which is the cutoff value for dementia screening, were excluded. Participants were also excluded if they had orthopedic conditions impairing pedal operation or if they failed to meet the visual and auditory standards required by Japanese road traffic laws, as self-reported. Ultimately, 48 older adults participated in the study, including 35 men and 13 women, ranging in age from 64 to 84 years (median = 73 years) and with a mean age of 73.5 years (SD = 4.9). The mean MMSE score was 28.3 points (SD = 1.5), with scores ranging from 25 to 30 points (median = 29 points). Regarding driving frequency, 28 participants reported driving daily, 19 drove several times a week to several times a month, and 1 drove less than once a month.

Young adults were recruited verbally from students at the university with which the authors were affiliated. For those who expressed interest, the study details were explained both in writing and verbally, and informed consent was obtained. The exclusion criteria for young adults included self-reported failure to meet the visual and auditory standards stipulated by Japanese road traffic laws. Ultimately, 56 young adults participated in the study, comprising 16 men and 40 women, ranging from 20 to 23 years (median = 21 years) and with a mean age of 21.1 years (SD = 0.7). Regarding driving frequency, 5 participants reported driving daily, 6 drove several times a week to several times a month, 33 drove less than once a month, and 12 did not hold a driver’s license. Compared to older adults, young adults exhibited a significantly lower driving frequency (*p* < 0.001). The demographic information of the participants is presented in Table 1.

### 2.2. Pedal Coordination Assessment Device

**Figure 1:**
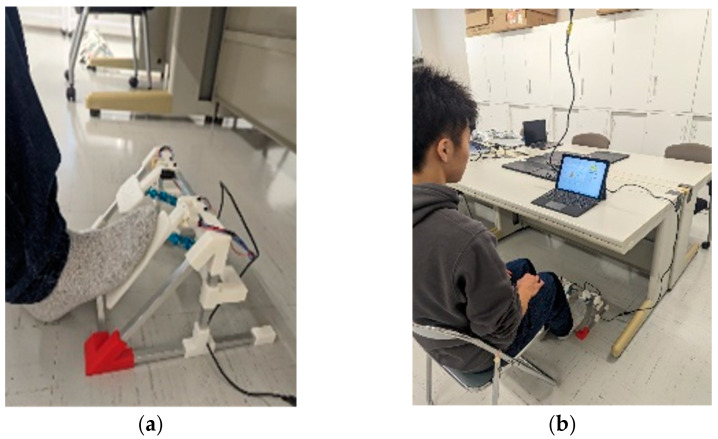
(**a**) The developed pedal device; (**b**) A participant undergoing the evaluation.

The developed pedal adjustment ability assessment device integrates a display tablet (Latitude 5285, Dell Technologies Inc., Round Rock, TX, USA) and a pedal apparatus into a single unit (Figure 1). The size and angle of the pedals were designed based on the specifications of actual automobiles and the G Controller (LPRC-15000d, Logitech International, Lausanne, Switzerland). Additionally, the pedal configuration follows the suspended pedal system, which is commonly used in vehicles driven by older adults in Japan. To achieve miniaturization and weight reduction, the device was designed with dimensions of 280 mm (width) × 180 mm (height) × 280 mm (depth). It is constructed using aluminum square tubing and poly-lactic acid (PLA) components, which were fabricated using a 3D printer. The pedal-to-floor angle is set at 50°, the height from the floor to the center of the pedal is 130 mm, and the maximum stroke distance is approximately 50 mm. For conditions requiring minimal or maximal pedal depression, the stroke range was set to approximately 20 mm, which corresponds to an ankle motion range of approximately 10°. The required pedal force for operation was set at approximately 10 N, based on the study by Weaver et al. [27]. The spring’s restoring force was adjusted to replicate the tactile feedback experienced when operating an actual vehicle pedal. Adjustments to the spring and damper settings were made based on feedback from licensed drivers who tested the device. Furthermore, an angle sensor was attached to the pedal’s rotational axis to measure the pedal depression. The output values from the angle sensor were used to compute speed using the following Equation (1):

V = C_A_ (θ_A_ − θ_A0_) − C_B_ (θ_B_ − θ_B0_) (1)

θ_A_ and θ_B_ represent the angle sensor values, and θ_A0_ and θ_B0_ indicate the initial values of the angle sensor. A and B denote the accelerator and brake pedals, respectively. The coefficients for speed and braking were experimentally determined based on the subjective perception of licensed drivers, with values set at C_A_ = 1.4 and C_B_ = 4.

A major advantage of the developed device is that it is lightweight, compact, and portable, allowing for simultaneous assessment of multiple individuals in various locations. Notably, the pedal system is not intended for installation in vehicles used for the on-road evaluation described later, nor in vehicles personally owned by participants. Instead, the device is exclusively designed for use in an indoor setting within the preventive care program.

### 2.3. Procedure

**Figure 2:**
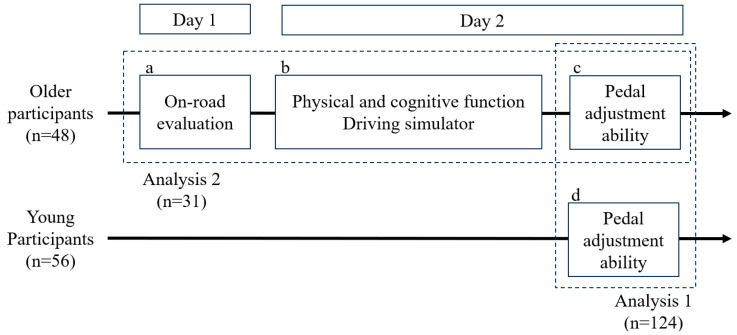
The research flowchart. Older adults underwent an on-road evaluation (**a**). On a separate day, they completed physical and cognitive function tests, a driving simulator assessment, and the pedal adjustment ability evaluation (**b**,**c**). Younger adults only participated in the pedal adjustment ability evaluation (**d**). For the analysis, a two-way ANOVA was conducted to verify whether the developed pedal adjustment ability evaluation device could differentiate between older and younger adults. The analysis included a total of 124 participants (Analysis 1; **c**,**d**). Subsequently, the correlation between the developed pedal device and driving-related abilities was examined. This analysis included 31 older adults, excluding those with missing data (Analysis 2; **a**–**c**).

First, the overall research flowchart is presented in Figure 2.

A total of 48 older adults underwent on-road evaluations conducted by driving instructors. Each evaluation was performed on a different day between 2:00 and 2:50 PM. The participants drove a designated route of 7–8 km (approximately 25–30 min) within the city, using a training vehicle from a driving school. During the evaluation, instructors provided advice on driving skills and assessed the participants’ driving abilities based on the Driving Skill Test Scorecard [28], a standardized evaluation form mandated by Japanese road traffic laws. The Driving Skill Test Scorecard includes 11 categories of demerit points, each comprising detailed criteria. The 11 categories assessed in the Driving Skill Test Scorecard are as follows:

1. Driving posture—e.g., the driver’s body is not facing forward in alignment with the steering wheel.
2. Safety checks—e.g., failure to directly check surroundings when starting or changing lanes or failure to signal when turning.
3. Braking—e.g., not moving the foot to the brake pedal despite road conditions requiring deceleration.
4. Steering—e.g., noticeable wavering while driving.
5. Vehicle control—e.g., inability to position the vehicle appropriately to avoid potential collisions with bicycles or motorcycles approaching from behind when making a left turn.
6. Lane usage—e.g., driving in prohibited areas.
7. Lane changes—e.g., failing to move the vehicle toward the left side of the lane several meters before making a left turn.
8. Straight driving and turning—e.g., failing to decelerate appropriately when making right or left turns.
9. Pedestrian protection—e.g., obstructing pedestrians attempting to cross the road.
10. Maximum speed—e.g., exceeding the posted speed limit.
11. Evaluation suspension—e.g., failing to comply with a stop sign.

The scoring system employed a point deduction method, in which a major problem was assigned a deduction of 20 points, a moderate problem 10 points, and a minor problem 5 points. The total score was then calculated by summing these deductions, resulting in a range from 100 (best) to no lower limit (worst). For the analysis, the total score from the Driving Skill Test Scorecard and deductions for each category were used as the evaluation indices. However, three categories—driving posture, lane usage, and pedestrian protection—were excluded because none of the participants incurred deductions in these areas (Appendix A). Additionally, evaluation suspension was excluded from the analysis because all instances of suspension among participants were due to failure to stop at stop signs. During the on-road driving session, an accelerometer (Eco-sam, Toward Co., Ltd., Kanzaki, Japan) was used to automatically measure the undulation driving index (indicating the smoothness of acceleration and braking, with 100 representing the ideal score) and the number of instances of rapid acceleration and deceleration. For the analysis, the following parameters were used: the undulation driving index and the number of rapid acceleration and deceleration events while driving at speeds less than 40 km/h and between 40 and 60 km/h. The undulation driving index for speeds between 60 and 80 km/h was excluded because only four participants drove in this speed range.

On a day separate from the on-road evaluation, additional assessments were conducted to measure the physical and cognitive functions of the older adults. Grip strength and the Short Physical Performance Battery (SPPB) (scored out of 12 points, with higher scores indicating better physical function) [29] were measured as indicators of physical function. The SPPB includes three assessments: balance test, gait speed test, and chair stand test. The balance test evaluates the ability to maintain a standing position for at least 10 s with one foot’s heel placed against the toes of the other foot. The gait speed test measures the time required to walk a 4-m distance. The chair stand test assesses lower limb strength by recording the time taken to complete five repetitions of standing up from and sitting back down on a chair. For cognitive function, participants completed the MMSE and the Japanese version of the Trail Making Test (TMT-J). The MMSE includes assessments of temporal and spatial orientation, immediate recall, arithmetic calculation, short-term memory, comprehension of instructions, and figure copying. The test has a maximum score of 30 points, with scores below 24 points indicating cognitive impairment. The TMT-J is a paper-based task in which participants connect numbers and characters with lines according to a specific rule. The completion time is recorded as the primary outcome measure. The test consists of two parts: Part A and Part B. Part A requires participants to sequentially connect numbers from 1 to 25 in ascending order. Part B involves alternately connecting numbers and Japanese hiragana characters in order (e.g., 1 → “あ” → 2 → “い” → 3 → “う”, and so on). Additionally, the participants performed tasks on a DS (manufactured by Honda, Safety-Navi, Tokyo, Japan), which is a Japanese-language simulator used in studies such as that by Nakagawa et al. [30]. The DS assessments included a driving reaction test and a course driving task (labeled as a “hazard prediction training course”). In the driving reaction test (a dual-task paradigm), participants were instructed to respond to visual cues displayed on a screen by pressing the accelerator or brake pedal. The outcome measures for this test included reaction time and number of incorrect responses. For the course-driving task, the participants drove the same 5-min course three times, with data from the third trial used for the analysis. The outcome measures for the course-driving task included the number of accidents and hard-braking events.

**Figure 3:**
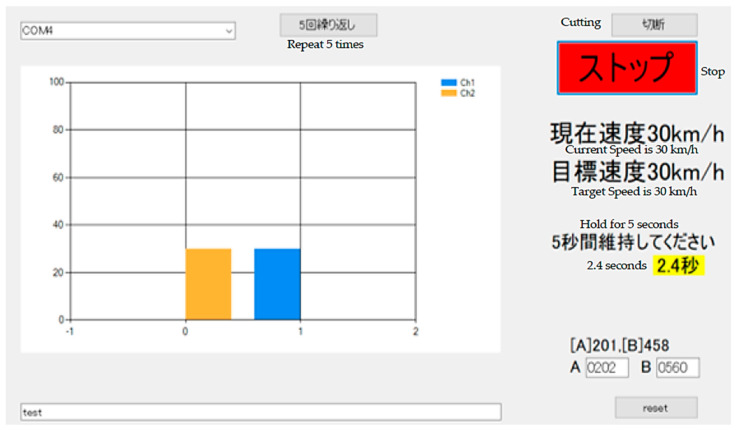
Screen displayed to participants. The figure shows the participant’s pedal speed (blue gauge on the right) being maintained at the target speed (orange gauge on the left) for 2.4 s (center-right of the figure).

Finally, the pedal adjustment ability of the participants was measured using the developed device. During the task, the display presented a target speed gauge at one of the four levels (30, 40, 60, or 70 km/h) (Figure 3, orange gauge on the left). The participants were instructed to press the accelerator pedal to align the pedal gauge (Figure 3, blue gauge on the right) with the target speed gauge. If the pedal position was maintained within a ±5 km/h margin of the target speed for 5 s, the next target speed gauge was presented. Figure 3 shows an example in which the pedal gauge aligns with the 30 km/h target speed gauge, with 2.4 s elapsed. The evaluation sequence involved repeating a set of four target speeds (30, 60, 40, and 70 km/h) in order for five sets. If the pedal gauge deviated from the margin before maintaining its position for 5 s, the timer was reset. A key feature of the task was that participants were required to respond to changes in the target gauge through pedal operation. For example, in the 30 km/h target condition, participants had to transition from the 70 km/h pedal position to the 30 km/h position by easing the pedal pressure and maintaining their ankle position for 5 s. For the analysis, the average time required to achieve 5 s of pedal stability across the second to fourth sets for each target speed condition was calculated as a representative value for each participant. The time required to achieve 5 s of pedal stability was used as an indicator of the pedal adjustment ability, with shorter times indicating higher ability. The data sampling frequency was set to 10 Hz. After completing the pedal task, participants were asked to rate the subjective difficulty of the task on a four-point scale (“easy”, “somewhat easy”, “somewhat difficult”, “difficult”).

Younger participants were assessed only for their pedal adjustment ability. The same device and task used for older participants were employed for this assessment.

### 2.4. Analysis

To validate the pedal adjustment ability assessment device, a two-way analysis of variance (ANOVA) was conducted with the time required to maintain pedal position for 5 s as the dependent variable. The independent variables were age group (older adults, younger adults) and target speed condition (30 km/h, 40 km/h, 60 km/h, 70 km/h). Additionally, subjective difficulty ratings were compared between age groups using a chi-square test (χ^2^). The significance level was set at 5%. Effect sizes, including η^2^, Cohen’s d, and Cramer’s V, were calculated to indicate the magnitude of the differences. The criteria for effect sizes were defined as follows, in increasing order of magnitude: small, medium, and large. Specifically, η^2^ was set at 0.01, 0.06, and 0.14, Cohen’s d at 0.2, 0.5, and 0.8, and Cramer’s V at 0.1, 0.3, and 0.5 [31,32].

To explore potential applications of the pedal adjustment ability assessment device, the correlations between pedal adjustment ability and physical function, cognitive function, and driving-related abilities were analyzed for older adults. Pearson correlation coefficients (r) and 95% confidence intervals were calculated for these analyses. The criteria for correlation strength were defined as small, medium, and large, corresponding to 0.1, 0.3, and 0.5, respectively [31]. Of the 48 older adults recruited, data from 31 participants (20 men and 11 women; mean age 75.6 ± 4.1 years, range 67–84 years, median 76 years) were included in the correlation analyses after excluding individuals with missing data.

## 3. Results

### 3.1. Pedal Adjustment Ability

**Figure 4:**
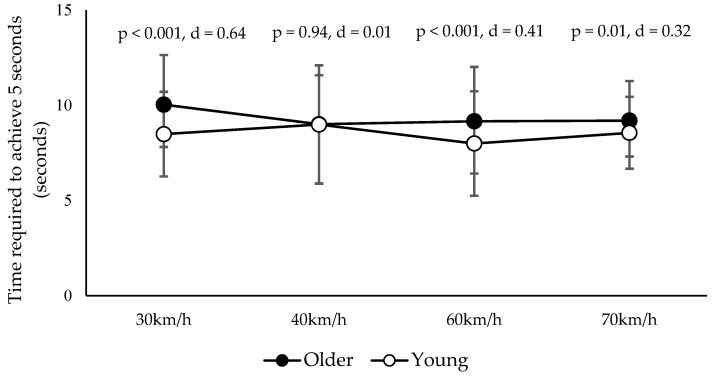
Results of pedal adjustment ability. Time (s) required to maintain the pedal for 5 s at each target speed for older adults and younger adults. *p*-values and effect sizes (Cohen’s d) for group comparisons (Tukey’s method) are shown.

The results of the two-way analysis of variance (ANOVA) showed no significant interaction effect (*p* = 0.07, η^2^ = 0.01) and no significant main effect within groups (*p* = 0.17, η^2^ = 0.01). Subsequently, to clarify the differences between older and younger participants, simple main effects and multiple comparison tests were performed using Tukey’s method. The analysis revealed that the older participants required significantly more time than the younger participants to maintain the pedal position for 5 s under certain conditions. The results were as follows: 30 km/h condition, *p* < 0.001, Cohen’s d = 0.64; 40 km/h condition, *p* = 0.94, Cohen’s d = 0.01; 60 km/h condition, *p* < 0.001, Cohen’s d = 0.41; and 70 km/h condition, *p* = 0.01, Cohen’s d = 0.32. These findings are summarized in Figure 4. In terms of subjective difficulty, the majority of participants in both groups reported the task as “somewhat difficult”. A chi-square test revealed no statistically significant differences between the groups (*p* = 0.34, Cramer’s V = 0.18).

A two-way ANOVA (gender × target speed) was conducted to investigate the influence of gender differences within the older participant group. The motivation for examining gender differences stems from previous reports on gender disparities in physical function [33], cognitive function [34], and driving ability [35] among older adults. The analysis revealed no significant interaction effect (*p* = 0.38, η^2^ = 0.02) and no significant main effect within groups (*p* = 0.52, η^2^ = 0.02).

Subsequently, simple main effects and multiple comparison tests using Tukey’s method were performed to clarify gender differences under each target speed condition. The results showed that for all target speeds, older women required significantly more time than older men to maintain the pedal position for 5 s. The results were as follows: 30 km/h condition, *p* = 0.03, Cohen’s d = 0.37; 40 km/h condition, *p* < 0.001, Cohen’s d = 1.18; 60 km/h condition, *p* = 0.003, Cohen’s d = 0.58; and 70 km/h condition, *p* = 0.002, Cohen’s d = 0.8. In terms of subjective difficulty, the majority of both older men and older women reported the task as “somewhat difficult”. A chi-square test revealed no statistically significant differences between genders (*p* = 0.12, Cramer’s V = 0.35).

### 3.2. Correlation Between Pedal Adjustment Ability and Physical, Cognitive, and Driving Ability

For physical and cognitive function measures, a moderate negative correlation was observed between grip strength and the 40 km/h target speed condition (r = −0.39, 95% CI [−0.66, −0.03]), and a significant moderate positive correlation was found between MMSE scores and the 60 km/h target speed condition (r = 0.5, 95% CI [0.16, 0.73]). Regarding driving ability indicators, there were moderate positive correlations between the number of accidents during the DS course-driving task and the 60 km/h (r = 0.52, 95% CI [0.11, 0.78]) and 70 km/h target speed conditions (r = 0.58, 95% CI [0.20, 0.81]). Additionally, a moderate positive correlation was observed between the number of sudden braking incidents and the 30 km/h target speed condition (r = 0.45, 95% CI [0.03, 0.74]). Conversely, the number of sudden accelerations during on-road driving showed a moderate negative correlation with the 30 km/h target speed condition (r = −0.59, 95% CI [−0.81, −0.22]). No significant correlations were found between pedal adjustment ability and the DS driving reaction task, on-road evaluation, or wave driving index during driving (Table 2).

## 4. Discussion

In this study, we examine the validity of a pedal adjustment ability evaluation device by comparing the pedal adjustment ability of older and younger adults. The results revealed that older adults exhibited poorer pedal adjustment ability than younger adults. Furthermore, among older adults, women demonstrated lower pedal adjustment ability than men. To explore the potential applications of the developed device, we analyzed the correlations between the pedal adjustment ability and physical, cognitive, and driving-related indicators in older adults. The results showed that the time required to maintain the pedal position for 5 s was positively correlated with the number of accidents and sudden braking incidents during the DS driving task as well as the number of sudden accelerations observed during the on-road evaluation. However, no negative correlations were observed between pedal adjustment ability and physical or cognitive function measures, wave driving indices, or braking performance during the on-road evaluation.

For older adults, recognizing the decline in their physical and cognitive function as well as driving ability is essential for adopting self-regulatory driving [14,15,16]. Additionally, providing opportunities to assess these functional and ability declines is a critical role of healthcare professionals involved in preventive care programs. In this study, the device that was developed successfully distinguished the pedal adjustment ability of older and younger adults, demonstrating its validity in reflecting age-related changes. Accurate task performance with this device requires proprioception of the ankle joint [36], coordination of the visual input with ankle movements, and synergistic activity of the muscles involved in ankle motion [37,38,39]. The findings of this study likely reflect the physiological decline in proprioceptive sensitivity and the deterioration of visuomotor coordination associated with aging. Moreover, despite older adults having a higher driving frequency than younger adults, their poorer performance suggests that driving frequency may not influence the pedal adjustment ability assessed by the developed device.

Age-related changes in physical and cognitive function, as well as driving ability, exhibit gender differences [33,34,35]. Similarly, gender differences have been reported in age-related changes in visuomotor coordination [40]. The developed device successfully distinguished pedal adjustment abilities between older men and women, indicating that it reflects not only the effects of aging but also gender differences. Furthermore, the absence of age- or gender-based differences in the subjective evaluations of the pedal task suggests the importance of using the developed pedal adjustment device to objectively help individuals recognize age-related decline in their abilities.

Considering the socially beneficial applications of the developed device is critically important [41]. We envision using this pedal coordination assessment device to guide the design and direction of programs provided as part of preventive care initiatives. Specifically, if pedal coordination ability is found to correlate with physical and cognitive function, it could inform the design of training content. Similarly, if pedal coordination ability is associated with on-road evaluations, it might serve as a reference for advising drivers on driving behaviors. Our anticipated outcome was that the time required to maintain the pedal position for 5 s would negatively correlate with physical, cognitive, and driving abilities. However, the only logically interpretable correlations observed were a positive correlation between the time required for pedal maintenance and both the number of crashes and instances of sudden braking in the DS and a negative correlation with grip strength. There were no significant associations with the demerit items from the on-road evaluations or the smoothness of pedal operation, as measured by the undulating driving index. Although these findings differ from our initial hypothesis, they may reflect the inherent difficulty of predicting real-world pedal operation using a simulated pedal device [42].

### Limitation

This study had several limitations. First, selection bias may have been an issue. The older adults who participated in this study were more likely to have higher physical and cognitive abilities than the general older adult population. For example, the median MMSE score was 29, and the median SPPB score was 12 (perfect score). Although this may have contributed to the robustness of the validity evaluation when comparing older adults with younger participants, it may have influenced the detection of correlations in the analyses. Second, there was an issue with time resolution when calculating the undulating driving index. In this study, the undulating driving index was averaged over a fixed time frame, such as the total time spent driving at speeds below 40 km/h, to evaluate the smoothness of pedal operation. However, abrupt pedal operation during the on-road evaluation may have been omitted during the averaging process. This limitation in the time resolution may have affected the correlation analyses. The third point concerns the WHO’s Health Technology Assessment (HTA) [43]. This study demonstrated the validity of the developed pedal device in distinguishing between younger and older adults. However, before the device can be widely implemented in public health policies, a comprehensive HTA should be conducted. HTA considers not only the device’s validity but also its cost-effectiveness, feasibility in different healthcare settings, long-term usability, and potential ethical and social implications. Additionally, large-scale studies are required to assess the device’s reliability across diverse populations and real-world conditions. Further research is needed to address these aspects, ensuring that the device meets the necessary standards for broader adoption in public health initiatives.

## 5. Conclusions

In this study, we developed a pedal adjustment ability assessment device designed for implementation in group-based preventive care programs. This device aims to enhance awareness of driving ability decline among older adults. To evaluate its validity, we compared the pedal adjustment ability between older and younger adults. Additionally, we analyzed the relationship between pedal adjustment ability and driving performance in older adults.

The results demonstrated that the device effectively distinguished between younger and older adults, indicating its validity in reflecting the effects of aging. Additionally, the limited correlation between pedal coordination ability and physical, cognitive, and driving-related capabilities suggests that the device should be primarily used as a tool to help individuals recognize the decline in their pedal coordination ability due to aging. Future research should focus on conducting group assessments within preventive programs and examining the impact of the device on promoting self-regulated driving among participants.

Finally, we believe that the pedal adjustment ability assessment device developed in this study is closely aligned with the goals of the SDGs Agenda 2030 [44]. Particularly Goal 3 (Ensure healthy lives and promote well-being for all at all ages) [45] by promoting active aging and preventing accidents through early self-awareness. Additionally, it supports Goal 11 (Make cities and human settlements inclusive, safe, resilient, and sustainable) [45] by contributing to safer mobility solutions for older adults. Further research and implementation efforts are needed to integrate this device into public health strategies, ensuring its accessibility and impact in promoting sustainable and safe mobility for aging populations.

## Figures and Tables

**Table 1:** Characteristics of participants. High driving frequency includes the number of individuals who drive daily or drive weekly/monthly, while low driving frequency includes those who rarely drive or never drive.

	Older (N = 48)	Young (N = 56)
age (range, median)	73.5 ± 4.9 (64–84, 73)	21.1 ± 0.7 (20–23, 21)
sex (male/female)	35/13	16/40
driving frequency (high/low)	47/1	11/45
MMSE (range, median)	28.3 ± 1.5 (25–30, 29)	-

MMSE; Mini-Mental State Examination.

**Table 2:** Correlation between pedal adjustment ability and physical, cognitive, and driving ability.

	Target Speed							
	30 km/h		40 km/h		60 km/h		70 km/h	
	r	[95%CI]	r	[95%CI]	r	[95%CI]	r	[95%CI]
Age	0.1	[−0.26, 0.44]	−0.10	[−0.44, 0.27]	0.11	[−0.26, 0.45]	0.25	[−0.12, 0.55]
[Physical, cognitive function]								
grip strength	−0.15	[−0.48, 0.22]	−0.39	[−0.66, −0.03]	−0.15	[−0.49, 0.22]	−0.20	[−0.52, 0.18]
SPPB	−0.15	[−0.48, 0.21]	−0.09	[−0.43, 0.28]	−0.17	[−0.50, 0.19]	−0.09	[−0.43, 0.27]
MMSE	−0.22	[−0.54, 0.16]	0.24	[−0.14, 0.55]	0.50	[0.16, 0.73]	0.26	[−0.12, 0.57]
TMT-A	0.21	[−0.17, 0.53]	−0.04	[−0.40, 0.33]	−0.21	[−0.53, 0.17]	0.30	[−0.08, 0.60]
TMT-B	0.17	[−0.21, 0.50]	−0.07	[−0.42, 0.31]	−0.07	[−0.42, 0.31]	0.28	[−0.10, 0.59]
[DS driving reaction test]								
RT	0.07	[−0.30, 0.42]	−0.23	[−0.54, 0.15]	−0.16	[−0.49, 0.21]	0.01	[−0.36, 0.36]
miss	−0.02	[−0.37, 0.35]	−0.14	[−0.47, 0.24]	−0.21	[−0.53, 0.17]	0.10	[−0.27, 0.44]
[DS course-driving task]								
accidents	0.10	[−0.34, 0.51]	0.34	[−0.11, 0.67]	0.52	[0.11, 0.78]	0.58	[0.20, 0.81]
rapid deceleration	0.45	[0.03, 0.74]	−0.14	[−0.54, 0.31]	−0.33	[−0.67, 0.12]	0.10	[−0.35, 0.51]
[On-road evaluation]								
total score	0.02	[−0.34, 0.37]	−0.12	[−0.46, 0.24]	−0.12	[−0.45, 0.25]	−0.16	[−0.49, 0.20]
safety checks	0.16	[−0.21, 0.48]	0.35	[−0.01, 0.63]	0.15	[−0.22, 0.47]	0.12	[−0.25, 0.45]
braking	0.07	[−0.29, 0.42]	−0.13	[−0.46, 0.24]	0.08	[−0.28, 0.43]	−0.12	[−0.45, 0.25]
steering	−0.30	[−0.59, 0.06]	−0.15	[−0.48, 0.22]	0.03	[−0.33, 0.38]	0.08	[−0.29, 0.42]
vehicle control	−0.07	[−0.41, 0.29]	0.10	[−0.27, 0.44]	0.02	[−0.34, 0.37]	0.14	[−0.23, 0.47]
lane changes	0.12	[−0.24, 0.46]	0.04	[−0.32, 0.39]	0.09	[−0.28, 0.43]	0.35	[0.0, 0.63]
straight driving and turning	−0.30	[−0.59, 0.06]	−0.12	[−0.45, 0.24]	−0.20	[−0.52, 0.16]	−0.09	[−0.43, 0.27]
maximum speed	0.11	[−0.26, 0.44]	0.00	[−0.36, 0.35]	0.22	[−0.15, 0.53]	0.02	[−0.34, 0.37]
[Undulation driving index]								
−40 km/h	−0.21	[−0.58, 0.23]	0.11	[−0.32, 0.51]	−0.16	[−0.55, 0.28]	0.02	[−0.40, 0.44]
40–60 km/h	0.09	[−0.35, 0.49]	0.05	[−0.38, 0.46]	−0.07	[−0.48, 0.36]	−0.12	[−0.52, 0.32]
rapid acceleration	−0.59	[−0.81, −0.22]	−0.20	[−0.57, 0.25]	−0.22	[−0.59, 0.22]	−0.28	[−0.62, 0.17]
rapid deceleration	−0.01	[−0.43, 0.41]	−0.10	[−0.50, 0.33]	−0.12	[−0.52, 0.32]	0.08	[−0.36, 0.48]

Note: Statistically significant correlations are underlined. r, Pearson’s correlation coefficient; 95%CI, 95% confidential interval [lower, upper]; SPPB, Short Physical Performance Battery; MMSE, Mini-Mental State Examination; TMT-A, Trail Making Test part A; TMT-B, Trail Making Test part B; RT, reaction time; ‘−40 km/h’ represents driving at speeds below 40 km/h, while ’40–60 km/h’ represents driving at speeds between 40 km/h and below 60 km/h.

**Figure A1:**
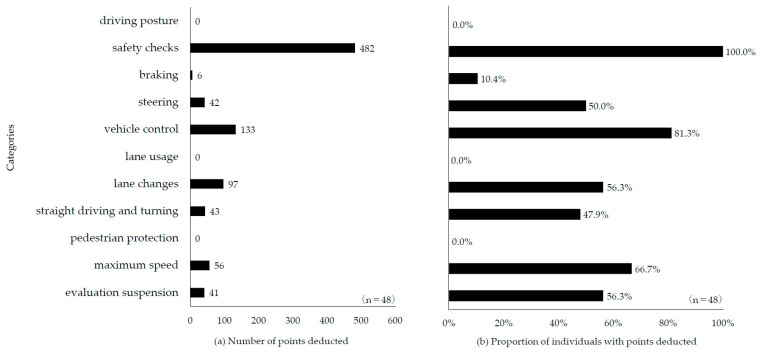
Results of the Driving Skill Test Scorecard from the on-road evaluation for 48 older adults. (**a**) The number of categories in which points were deducted during the on-road evaluation. (**b**) The proportion of older adults (out of 48) who had at least one deduction in each category. Safety checks had the highest number of deductions, with all older adults receiving point deductions. While the number of deductions for maximum speed and evaluation suspension (failure to stop) was not high (**a**), more than half of the older adults were penalized in these categories (**b**). The average Driving Skill Test Scorecard score was −79.1 ± 83 points (minimum: −330, maximum: 50, median: −70). The older adults in the study by Shimada et al. [28] had an average score of approximately −130 ± 50 points.

## Data Availability

The original contributions presented in this study are included in the article. Further inquiries can be directed to the corresponding author.
